# Occupational risk perception of construction workers: a cross sectional study

**DOI:** 10.3389/fpubh.2024.1338604

**Published:** 2024-01-26

**Authors:** Riccardo Mastrantonio, Vincenza Cofini, Giada Mastrangeli, Mauro Pettinaro, Marianna Mastrodomenico, Leila Fabiani

**Affiliations:** ^1^Department of Life, Health and Environmental Sciences, University of L’Aquila, L’Aquila, Italy; ^2^CGIL Trade Union, Teramo, Italy; ^3^Local Health Authority of Avezzano-Sulmona-L’Aquila, L’Aquila, Italy

**Keywords:** construction industry, building site, occupational injury, occupational risk, risk perception, work injury perception, construction worker, occupational hazard

## Abstract

**Introduction:**

Construction workers are often exposed to significant injury risk. The aim of our study is to assess their occupational hazards and injury risk perception.

**Methods:**

We administered 256 questionnaires to construction workers. The survey was aimed at collecting information regarding occupational risk and hazard exposure perception, risk control and behavioral self-assessment. We analyzed the data obtained in order to highlight any associations between injury risk perception and anamnestic, occupational, behavioral or perceptual factors.

**Results:**

Participants were prevalently males (92.37%) aged 21–60 years (94%). They showed a job seniority level of 21.3 (11.51) on average and, ranging from a 1 to 10 score, a danger awareness of 6.8 (2.9), a lack of prevention measures 6.0 (3.3), an improper behavior of 7.3 (2.7), an unpredictable fate of 6.0 (2.9). These factors resulted significantly associated with the injury risk perception. Multivariable analysis highlighted that the injury risk perception was associated with the lack of prevention measure and unpredictable fate. On the other hand, we found a negative association with the workers’ improper behaviors.

**Conclusion:**

Workers’ perception showed fairly uniform average values even when occupational risk was demonstrated. Our analysis suggests a positive correlation between injury risk perception and the idea that injuries are due both to fate and to chance; it also shows a negative correlation between injury risk perception and the idea that injuries are due to improper behavior. A lack of fully comparable studies confirms the need for further studies on the injury risk perception of construction workers.

## Introduction

In 2009 a disastrous earthquake struck the city of L’Aquila in central Italy; it caused 309 deaths, and it destroyed a huge number of buildings: the reconstruction involved thousands of buildings (1).

During the first phase of the emergency, 70,000 inhabitants were evacuated from the area which was repeatedly hit by successive shocks. Public safety and public psychophysical health were a major challenge for the authorities and the national health system that had to take care of the population who suffered the effects of the earthquake (2–4). The damaged area involved more than 80 villages spread across 57 municipalities. (5, 6). The reconstruction of private and public buildings started in 2009; to date, in 2023, the reconstruction of the city has not yet been completed (7).

In accordance with the European regulations, behaving properly in workplace and respecting occupational health regulatory measures is functional to prevent accidents and diseases (8). Despite the governmental supervision and the Italian safety rules, the Italian National Institution for Occupational Accident Insurance recorded more than a half million accidents at work in 2021 (9).

The construction industry is one of the most dangerous businesses for the safety and health of workers. Indeed, construction workers suffer from many work-related illnesses and are subject to work-related injuries (10–13).

Risk perception is one of the factors that may strongly influence safety in the workplace: workers have to constantly evaluate and judge the hazards to which they might be exposed. Work-related injuries are usually caused by unsafe working conditions and risk-taking behaviors (14, 15).

The aim of our study was to investigate the occupational risk perception (occupational risk, hazard exposure, risk control and behavioral self-assessment) in workers engaged in reconstruction in L’Aquila (Italy), with a focus on the injury risk perception and its associations with the job activities.

## Materials and methods

The study was a cross-sectional study, authorized by the Ethical Committee (EC) of the Local Health Authority Avezzano-Sulmona-L’Aquila, Abruzzo Region, Italy (minutes dated 21 June 23, 2021). Our project involved the voluntary participation of construction companies engaged in the city reconstruction. The companies were reached out through informal channels by the Joint Local Committee and the Local Health Authority; 6 construction companies took part in the project.

Data were collected from June 2021 to June 2022 by administering a questionnaire (Supplementary material) and self-reported data were digitally stored.

Questionnaires were completed during the working activities at the construction sites. Informed consent from all the participants was obtained; confidentiality and anonymity have been guaranteed.

In accordance with the aim of the study, the questionnaire included items about medical history (pre-existing diseases); habits and lifestyle (smoking and alcohol consumption); job information (job task, seniority; Table 1). It also investigated the following dimensions:

- Occupational risk and hazard exposure (10 items showed in Table 2); 5 items allow three possible answers (At all/Moderately/Highly) and the remaining 5 items allow a 0 to 10 answer scale. These dimensions are investigated by asking an opinion about occupational risk and hazard exposure;
- Risk control and behavioral self-assessment; (11 items showed in Table 3); 6 items allow three possible answers (At all/Moderately/Highly or Lacking/Enough/Good or Never/Sometimes/Always); 1 item allows a dichotomous answer (yes/no), and the remaining 4 items allow a 0 to 10 answer scale. These dimensions are investigated by asking an opinion about the chance of being injured and about job characteristics and related aspects.

**Table 1:** Descriptive data analysis.

Variables	Characteristic	*n* (%) or mean (SD)
Age (classes)	18–20	6 (2.76%)
	21–30	38 (17.51%)
	31–40	58 (26.72%)
	41–50	56 (25.80%)
	51–60	52 (23.96%)
	>61	7 (3.22%)
Sex	Male	109 (92.37%)
	Female	9 (7.63%)
Marital status	Married	133 (59.11%)
	Unmarried	55 (24.44%)
	Widow	1 (0.44%)
	Divorced	10 (4.44%)
	Cohabitant	26 (11.56%)
Educational attainment	Elementary school	17 (8.33%)
	Junior high school	74 (36.27%)
	High school	97 (47.55)
	University	16 (7.84%)
Nationality	Italian	165 (78.20%)
	Extra EU	10 (4.74%)
	EU	36 (17.06%)
Job task *	Specialized laborer	95 (45.89%)
	Laborer	48 (23.19%)
	Driver	22 (10.63%)
	Manager	19 (9.18%)
	Unemployed	23 (11.11%)
Seniority (years)	(free filling)	20.61 (11.51)
Have you ever worked in dusty environments?	yes	217 (88.21%)
	no	29 (11.79%)
Do you have heart conditions?	yes	3 (4.29%)
	no	67 (95.71%)
Do you have seizures?	yes	0 (0%)
	no	70 (100%)
Do you have lung conditions?	yes	2 (2.82%)
	no	69 (97.18%)
Do you have allergic rhinitis?	yes	6 (8.57%)
	no	64 (91.43%)
Do you have bronchial asthma?	yes	2 (2.86%)
	no	68 (97.14%)
Do you have bronchitis for more than 2 months a year?	yes	4 (5.71%)
	no	66 (94.29%)
Do you usually cough when you get up in the morning?	yes	20 (8.16%)
	no	225 (91.84%)
Do you need to catch your breath while walking?	yes	9 (3.67%)
	no	236 (96.33%)
Do you have shortness of breath while you are resting?	yes	10 (4.10%)
	no	234 (95.90%)
Have you had tetanus vaccination?	yes	71 (94.67%)
	no	4 (5.33%)
How many coffees do you drink a day?	(free filling)	3 (2)
Do you usually drink beer?	yes	58 (60.42%)
	no	38 (39.58%)
Do you usually drink wine?	yes	47 (47.96%)
	no	51 (52.04%)
Do you usually drink liqueurs?	yes	20 (22.22%)
	no	70 (77.78%)
Do you smoke?	yes	145 (57.09%)
	no	109 (42.91%)
Are you an ex-smoker?	yes	48 (28.24%)

*1 = Specialized laborer (Bricklayer, scaffolder, carpenter); 2 = Laborer (manual laborer, laborer, apprentice); 3 = Driver (driver, crane user); 4 = Management worker (surveyor, operations manager, employer, accountant, site manager, safety worker, architect, employee); 5 = Unemployed.

**Table 2:** Job harmfulness and hazard exposure self-assessment.

Variables	Worker assessment	*n* (%) or mean (SD)
How harmful do you consider your posture in your current job?	At all	55 (28.49%)
	Moderately	93 (48.18%)
	Highly	45 (23.31%)
How harmful do you consider manual handling in your current job?	At all	54 (27.83%)
	Moderately	88 (45.36%)
	Highly	52 (26.80%)
How harmful do you consider the climatic factors in your current job?	At all	61 (30.96%)
	Moderately	83 (42.13%)
	Highly	53 (26.90%)
How harmful do you consider strain in your current job?	At all	57 (29.84%)
	Moderately	85 (44.50%)
	Highly	49 (25.65%)
How harmful do you consider work organization in your current job?	At all	70 (37.03%)
	Moderately	74 (39.15%)
	Highly	45 (23.80%)
How exposed are you to physical risks?	mean (SD)	6.1 (2.8)
	median (range)	6 (0–10)
How exposed are you to chemical risks?	mean (SD)	4.4 (3.4)
	median (range)	4 (0–10)
How exposed are you to biological risks?	mean (SD)	3.9 (3.3)
	median (range)	4 (0–10)
How exposed are you to ergonomic risks?	mean (SD)	5.4 (3.1)
	median (range)	6 (0–10)
How exposed are you to work-related stress?	mean (SD)	5.0 (3.1)
	median (range)	5 (0–10)

**Table 3:** Risk control and behavioral self-assessment.

Variables	Worker assessment	*n* (%) or mean (SD)
Do you think your job is dangerous?	At all Moderately Highly	59 (26.10%) 98 (43.36%) 69 (30.53%)
Do you consider injuries a relevant danger in your job?	At all Moderately Highly	77 (36.84%) 91 (43.54%) 41 (19.61%)
How do you evaluate the training and the information received in relation to the risks you are exposed to?	Lacking Enough Good	4 (1.79%) 76 (33.93%) 144 (64.29%)
Do you think your behavior is appropriate to control occupational risks?	Never Sometimes Always	2 (0.88%) 60 (26.43%) 165 (72.69%)
Do you think you can rely on your employer’s protection?	Never Sometimes Always	3 (1.33%) 44 (19.47%) 179 (79.20%)
Do you think you can manage your job-related occupational risks?	Never Sometimes Always	4 (1.77%) 83 (36.72%) 139 (61.50%)
To what extent do you think a lack of knowledge and awareness of danger may lead to an injury?	mean (SD) median (range)	6.8 (2.9) 8 (0–10)
To what extent do you think a lack of prevention measures may lead to an injury?	mean (SD) median (range)	6.0 (3.3) 7 (0–10)
To what extent do you think an improper behavior in workplaces may lead to an injury?	mean (SD) median (range)	7.3 (2.7) 8 (0–10)
To what extent do you think unpredictable fate may lead to an injury?	mean (SD) median (range)	6.0 (2.9) 6 (0–10)
Do you think you have health and safety duties as a worker?	Yes No	158 (98.75%) 2 (1.25%)

All variables were analyzed and reported as frequencies or mean with standard deviations (SD). The Chi-Square test or T test for independent samples were used to compare categorical or continuous variables. The injury risk perception of the working activities (yes/no) was analyzed with univariate analysis with respect to the following factors: seniority, age (three classes: 18–30; 31–50; ≥50), Italian nationality (yes/no), educational attainment (high: high school or graduation/low: elementary or junior high school) marital status (cohabitant/not cohabitant), adequacy of training and information received (yes: if good /no: if lacking or enough), behavioral adequacy in order to control risks (yes: always/no: never or sometimes), employer protection (yes: always/no: never or sometimes), job-related occupational risk control (yes: always/no: never or sometimes), job task (laborer/driver/ manager), all factors that may lead to an injury: knowledge and awareness of danger (score), lack of prevention measures (score), improper behavior in workplace (score), unpredictable fate (score).

As showed in Table 4, the univariate analysis highlighted that some factors are significantly associated with the injury risk perception. The significant factors from Table 4 were entered into a multiple logistic reporting adjusted odds ratio (AOR) and relative 95% confidential intervals (95%CI).

**Table 4:** Injury-related danger perception and associated factors.

	Do you consider injuries a relevant danger in your job? (*n* = 209)		
	yes	no	p*
	Mean (SD) or *N* (%)	Mean (SD) or *N* (%)	
Seniority	21.3 (10.9)	16.5 (11.1)	0.0115
Age			
18–30	23 (57.5%)	17 (42.5%)	0.675
31–50	64 (64.65%)	35 (35.35%)	
>50	29 (65.91%)	15 (34.09%)	
Nationality			
Not Italian	25 (59.52%)	17 (40.48%)	0.596
Italian	89 (64.03%)	50 (35.97%)	
Educational attainment			
Low	46 (64.79%)	25 (35.21%)	0.991
High	55 (64.71%)	30 (35.29%)	
Marital status			
Cohabitant	85 (64.89%)	46 (35.11%)	0.239
Not cohabitant	33 (55.93%)	26 (44.07%)	
Do you think your job is dangerous?			
No	37 (24.18%)	35 (77.78%)	0.000
Yes	116 (75.82%)	10 (22.22%)	
How do you evaluate the training and the information received in relation to the risks you are exposed to?			
Inadequate	48 (65.75%)	25 (34.25%)	0.991
Adequate	79 (65.83%)	41 (34.17%)	
Do you think your behavior is appropriate to control occupational risks?			
No	35 (62.5%)	21 (37.5%)	0.536
Yes	94 (67.14%)	46 (32.86%)	
Do you think you can rely on your employer’s protection?			
No	27 (65.85%)	14 (34.15%)	0.974
Yes	101 (65.58%)	53 (34.42%)	
Do you think you can manage your job-related occupational risks?			
No	47 (65.28%)	25 (34.72%)	0.662
Yes	82 (68.33%)	38 (31.67%)	
Job task			
Laborer	91 (65.94%)	47 (34.06%)	0.967
Driver	9 (64.29%)	5 (35.71%)	
Management	12 (63.16%)	7 (36.84%)	
To what extent do you think a lack of knowledge and awareness of danger may lead to an injury?	7.30 (2.48)	6 (3.41)	0.0126
To what extent do you think a lack of prevention measures may lead to an injury?	6.75 (2.91)	5.03 (3.54)	0.0030
To what extent do you think an improper behavior in workplaces may lead to an injury?	7.69 (2.19)	6.70 (3.35)	0.0425
To what extent do you think unpredictable fate may lead to an injury?	6.93 (2.29)	4.96 (3.47)	0.0001

## Results

### Population characteristics

Overall, 226 workers were enrolled and filled out the questionnaire. As reported in Table 1, about half of them aged 31–50 years and 70.67% (159) were married or cohabitant. As expected, they were predominately males, and their nationality was mainly Italian (78.2%); most of the sample had a junior high school or a high school study degree (83.82%).

The average seniority was 20.61 years (11.51) and the identified job tasks were specialized laborer (45.89%), laborer (23.19%), driver (10.63%), manager (9.18%), and temporarily unemployed (11.11%).

Two hundred and seventeen participants reported working in dusty environments (88.21%). Of those reporting respiratory symptoms, the minority of the respondents reported having heart conditions (4.29%), lung conditions (2.82%), allergic rhinitis (8.57%), bronchial asthma (2.86%), and bronchitis (5.71%). None of the respondents reported having seizures. About the respiratory symptoms, 8.16% of the participants reported cough in the morning, 3.67% reported shortness of breath while walking, and 4.10% reported shortness of breath while resting.

Almost the whole sample reported being vaccinated against tetanus (94.67%). About alcohol consumption, 60.42% of workers reported drinking beer, 47.96% reported drinking wine, and 22.22% reported drinking liqueurs. Coffee consumption averaged 3 cups per day.

Regarding tobacco use, 145 workers reported smoking (57.09%); 28.24% of the sample reported being former smokers.

### Job harmfulness and occupational risks self-assessment

Almost half of the participants thought that posture is moderately harmful in their current job (48.18%); similar results were obtained concerning manual handling (45.36%), climatic factors (42.13%) and muscular strain (44.50%).

Scoring from 1 to 10, exposure to physical, chemical, biological, ergonomic, and work-related stress risk factors were perceived to be 6.13 (2.79), 4.4 (3.4), 3.9 (3.3), 5.44 (3.06) and 5.0 (3.1) on average, respectively. The results are shown in Table 2.

We assessed construction workers’ opinions about the hazard level, risk control, injuries, and organizational issues, such as safety training programs (Table 3).

Regarding the job dangerousness, more than a quarter of the participants (26.10%) consider their job not dangerous at all. Most respondents (64.29%, n. 144) assigned a “good” rating to the information and training received, they think to always be able to control occupational risks (61.50%, n.139) and to always behave correctly (72.69%, n. 165). Only 3 (1.33%) report a lack of protection by the employer with regard to occupational risks.

Overall, 98.75% think to have the duty to take charge of their health and safety as workers.

When exploring the most common determinants of injuries, the highest overall rating referred to improper behavior (7.3, SD 2.7) and a lack of knowledge and awareness (6.8, SD 2.9), with less concern for the shortfalls in preventive measures and unpredictable events.

### Workers perception

Analysis focused on the relationship between the injury risk perception, some personal and occupational characteristics, and individual risk perception (Table 4).

We observed a higher seniority among workers who consider injuries a real risk (*p* = 0.0115). We also found that danger awareness (*p* = 0.0126), a lack of prevention measures (*p* = 0.0030), improper behavior (*p* = 0.0425) and unpredictable fate (*p* = 0.0001) are factors associated with the injury risk perception.

Table 5 reports the significant factors associated with the injury risk perception as a result of multivariable analysis. Injury risk perception was higher in those who believe that a lack of prevention measures and unpredictable fate may lead to an injury; injury risk perception resulted positively associated with lack of prevention measure (AOR =1.28; 95% CI: 1.0407–1.5867) and unpredictable fate (AOR = 1.38; 95% CI: 1.0954–1.7479). Injury risk perception is lower in those who believe that improper behavior at work may cause accidents; in fact, it was negatively associated with the workers improper behaviors (AOR = 0.68; 95% CI:0.48620–0.95738).

**Table 5:** Multivariable logistic model: the injury risk perception dependent variable.

Independent factors	Odds ratio	Std. Err.	*p* > z	95% CI
Seniority	1.036938	0.0241218	0.119	0.99072–1.0853
To what extent do you think unpredictable fate may lead to an injury?	0.9993717	0.1289866	0.996	0.77600–1.2870
To what extent do you think a lack of prevention measures may lead to an injury?	1.285057	0.1382667	0.020	1.0407–1.5867
To what extent do you think an improper behavior in workplaces may lead to an injury?	0.6822627	0.117934	0.027	0.48620–0.95738
To what extent do you think unpredictable fate may lead to an injury?	1.38378	0.1649568	0.006	1.0954–1.7479

## Discussion

The main objective of our study focuses on two aspects: work-related risk perception and risk perception of occupational injuries. According to the data reported in Table 2, construction workers consider not harmful and moderately harmful the activities they carry out with reference to posture (76.67%), manual handling (73.19%), climatic factors (73.09%).

Despite these perceptions, we point out that risk assessment underscores the presence of postural, manual handling and climate-related dangers in the construction industry (16–18).

As reported in Table 2, the construction workers we interviewed perceive their job not so harmful regarding muscular strain (not harmful at all 29.84% and moderately harmful 44.50%). With regard to work organization, only 23.80% of workers consider it to be highly harmful.

The activities performed in a construction site can cause significant physical strain to workers. Yang et al. (19) found perceptions of muscular strain increased along with exposure time, workload, and temperature.

Regarding work organizations, results revealed, on average it may influence the health and wellbeing of construction workers (20, 21).

As shown in Table 2, perception of workers was, on average, low. This perception contradicts the findings reported in the scientific literature (13, 22, 23). Our interviewees assigned the highest mean value to physical risks exposure and the lowest to biological risks. With regard to biological hazards, a recent study carried out in Ghana showed that most of the biological hazards were perceived to be of medium-low importance level (24). This finding confirms what emerged from our survey.

In addition, physical, chemical and biological exposures among construction workers are non-negligible and are well described in the Scientific Literature.

It is demonstrated that building activities require non-neutral trunk postures (25), and that the work-related stress exposure, may lead to several physiological reactions (26, 27).

As reported by Boakye et al. (24), construction workers may show a low perception of occupational hazards: in accordance with our findings, chemical, biological and physical hazards were underestimated and resulted to be on central values overall. With regard to ergonomic hazard perception, despite our results suggest an underestimation, Boakye et al. reported a better perception of this sort of risk (24).

Work behavior is a major determinant of occupational health: 90% of the accidents on construction sites are caused by human errors (28) and are underlined by workers’ unsafe behaviors (29–31).

According to data reported in Table 3, in order to control occupational risks, 72.69% of the respondents deemed that their behavior is always correct, and 61.50% think to always be able to handle the occupational risks associated to their jobs.

Despite the construction industry being more dangerous than manufacturing industry (32), our participants considered working on construction sites moderately dangerous (43.36%), and considered injuries not a highly relevant danger.

With regard to the impact of human error, accidents involving the lack of awareness and incorrect assumptions relating to a dangerous situation, account for 49% of construction accidents (33). In our study, when asked about opinions and beliefs about injuries causes, on a scale from 0 to 10, inappropriate behavior in the workplace results as the most scored factor related to injury (mean score = 7.3 (2.7)), followed by lack of understanding and awareness about the danger (mean score = 6.8 (2.9)). Unsafe conditions, such as a lack of prevention measures, and unpredictable events appear to be less concerning than unsafe behaviors. Older workers mostly defined occupational accident experience as something unforeseen, younger and lower seniority workers defined it as an event caused by organizational factors. This is relevant because employees who believe that accidents are unavoidable and due to misfortune will have a reduced ability to take precautionary action (34).

The general trend shows a widespread underestimation of the occupational risks among construction workers.

We linked the characteristics of the sample and self-reported risk management to the perception of work-related injury (Do you consider injuries a relevant danger in your job? - Table 4). Multivariate analysis showed that the perception of work-related injuries is a factor associated with job seniority (*p* = 0.0115), with lack of knowledge (*p* = 0.0126), with lack of prevention measures (*p* = 0.0030), with improper behavior in workplaces (*p* = 0.0425), and with unpredictable fate (*p* = 0.0001).

These results were not all confirmed in multiple regression analysis (Table 5); in fact, after controlling for all factors reported in Table 4, the analysis showed that only three factors were associated with the perceived risk of work-related injury. It was positively associated with a lack of prevention measures (AOR = 1.28; 95% CI:1.0407–1.5867) and unpredictable fate (AOR = 1.38; 95% CI:1.0954–1.7479) but it was negatively associated with workers’ improper behaviors (AOR = 0.68; 95% CI:0.48620–0.95738).

It is possible to summarize what highlighted in the above, affirming that a general risk underestimation by construction workers exists. Workers’ perceived level of risk was uniformly middle low value even when the hazards in construction sites are widely demonstrated. In conclusion, our multiple regression analysis suggests a positive correlation between injury risk perception and the idea that injuries are due both to fate and to inadequate prevention measures. On the other hand, the analysis highlights a negative correlation between injury risk perception and the idea that injuries are due to improper behavior: this points out that workers underestimate the key role of correct behavior on occupational safety.

Our survey is based on a self-compiled questionnaire; this aspect represents a limitation, and it can lead to a significant possibility of recall and response bias. In addition, this led to missing responses with a lower number of analyzed items in our multiple regression analysis. A further limitation is due to the lack of fully comparable studies.

Construction workers underestimate occupational risks exposure and the work-related injury risk. In the light of these aspects, some actions to improve risk perception should be taken into account such as task-specific training and strategies to enhance risk knowledge. Although the specific, mandatory, and frequent training courses are positively evaluated by the group analyzed, a general risk and danger underestimation was found. The lack of fully comparable studies encourages us to increase the sample size and to expand on the current study with further research about risk perception among construction workers in Italy. Administering a similar survey to different industrial workers should also be interesting.

## Data availability statement

The raw data supporting the conclusions of this article will be made available by the authors, without undue reservation.

## Ethics statement

The studies involving humans were approved by Ethical Committee (EC) of the Local Health Authority Avezzano-Sulmona-L’Aquila. The studies were conducted in accordance with the local legislation and institutional requirements. The participants provided their written informed consent to participate in this study.

## Author contributions

RM: Conceptualization, Data curation, Methodology, Resources, Software, Visualization, Writing – original draft. VC: Data curation, Formal analysis, Visualization, Writing – review & editing. GM: Investigation, Methodology, Writing – original draft. MP: Data curation, Methodology, Writing – original draft. MM: Writing – review & editing. LF: Conceptualization, Formal analysis, Funding acquisition, Investigation, Methodology, Supervision, Validation, Writing – review & editing.
