# Supplementary material for: Occupational risk perception of construction workers: a cross sectional study
Source: Front Public Health. 2024 Jan 26;12:1338604. doi: 10.3389/fpubh.2024.1338604 (PMC10853437; doi:10.3389/fpubh.2024.1338604)
Supplement: Supplementary file 1 [file Table_1.DOCX]

Supplementary material: Self-reported questionnaire

| Age | ……………………… | | | | | | | | | |
| --- | --- | --- | --- | --- | --- | --- | --- | --- | --- | --- |
| Sex | - Male - Female | | | | | | | | | |
| Marital status | - Married - Unmarried - Widow - Divorced - Cohabitant | | | | | | | | | |
| Educational attainment | - Elementary school - Junior high school - High school - University | | | | | | | | | |
| Nationality | - Italian - Extra EU - EU | | | | | | | | | |
| What’s your job task now? | …………………………. | | | | | | | | | |
| Seniority | …………………………. | | | | | | | | | |
| Have you ever worked in dusty environments? | - yes - no | | | | | | | | | |
| Do you have heart conditions? | - yes - no | | | | | | | | | |
| Do you have seizures? | - yes - no | | | | | | | | | |
| Do you have lung conditions? | - yes - no | | | | | | | | | |
| Do you have allergic rhinitis? | - yes - no | | | | | | | | | |
| Do you have bronchial asthma? | - yes - no | | | | | | | | | |
| Do you have bronchitis for more than two months a year? | - yes - no | | | | | | | | | |
| Do you usually cough when you get up in the morning? | - yes - no | | | | | | | | | |
| Do you need to catch your breath while walking? | - yes - no | | | | | | | | | |
| Do you have shortness of breath while you are resting? | - yes - no | | | | | | | | | |
| Have you had tetanus vaccination? | - yes - no | | | | | | | | | |
| How many coffees do you drink a day? | ………………………. | | | | | | | | | |
| Do you usually drink beer? | - yes - no | | | | | | | | | |
| Do you usually drink wine? | - yes - no | | | | | | | | | |
| Do you usually drink liqueurs? | - yes - no | | | | | | | | | |
| Do you smoke? | - yes - no | | | | | | | | | |
| Are you an ex-smoker? | - Yes - no | | | | | | | | | |
| How harmful do you consider your posture in your current job? | - At all - Moderately - Highly | | | | | | | | | |
| How harmful do you consider manual handling in your current job? | - At all - Moderately - Highly | | | | | | | | | |
| How harmful do you consider the climatic factors in your current job? | - At all - Moderately - Highly | | | | | | | | | |
| How harmful do you consider strain in your current job? | - At all - Moderately - Highly | | | | | | | | | |
| How harmful do you consider work organization in your current job? | - At all - Moderately - Highly | | | | | | | | | |
| How exposed are you to physical risks? | 1 | 2 | 3 | 4 | 5 | 6 | 7 | 8 | 9 | 10 |
| How exposed are you to chemical risks? | 1 | 2 | 3 | 4 | 5 | 6 | 7 | 8 | 9 | 10 |
| How exposed are you to biological risks? | 1 | 2 | 3 | 4 | 5 | 6 | 7 | 8 | 9 | 10 |
| How exposed are you to ergonomic risks? | 1 | 2 | 3 | 4 | 5 | 6 | 7 | 8 | 9 | 10 |
| How exposed are you to work-related stress? | 1 | 2 | 3 | 4 | 5 | 6 | 7 | 8 | 9 | 10 |
| Do you think your job is dangerous? | - At all - Moderately - Highly | | | | | | | | | |
| Do you consider injuries a relevant danger in your job? | - At all - Moderately - Highly | | | | | | | | | |
| How do you evaluate the training and the information received in relation to the risks you are exposed to? | - Lacking - Enough - Good | | | | | | | | | |
| Do you think your behaviour is appropriate to control occupational risks? | - Never - Sometimes - Always | | | | | | | | | |
| Do you think you can rely on your employer’s protection? | - Never - Sometimes - Always | | | | | | | | | |
| Do you think you can manage your job-related occupational risks? | - Never - Sometimes - Always | | | | | | | | | |
| To what extent do you think a lack of knowledge and awareness of danger may lead to an injury? | 1 | 2 | 3 | 4 | 5 | 6 | 7 | 8 | 9 | 10 |
| To what extent do you think a lack of prevention measures may lead to an injury? | 1 | 2 | 3 | 4 | 5 | 6 | 7 | 8 | 9 | 10 |
| To what extent do you think an improper behaviour in workplaces may lead to an injury? | 1 | 2 | 3 | 4 | 5 | 6 | 7 | 8 | 9 | 10 |
| To what extent do you think unpredictable fate may lead to an injury? | 1 | 2 | 3 | 4 | 5 | 6 | 7 | 8 | 9 | 10 |
| Do you think you have health and safety duties as a worker? | - Yes - No | | | | | | | | | |
